# MicroRNA-224 sustains Wnt/β-catenin signaling and promotes aggressive phenotype of colorectal cancer

**DOI:** 10.1186/s13046-016-0287-1

**Published:** 2016-01-29

**Authors:** Tingting Li, Qiuhua Lai, Shuyang Wang, Juanjuan Cai, Zhiyuan Xiao, Danling Deng, Liuqing He, Hongli Jiao, Yaping Ye, Li Liang, Yanqing Ding, Wenting Liao

**Affiliations:** Department of Pathology, Nanfang Hospital, Southern Medical University, Guangzhou, 510515 Guangdong China; Department of Pathology, School of Basic Medical Sciences, Southern Medical University, Guangzhou, Guangdong China; State Key Laboratory of Oncology in Southern China, Department of Experimental, Guangzhou, Guangdong China

**Keywords:** miR-224, Proliferation, Invasion, Colorectal cancer, Wnt/β-catenin, GSK3β, SFRP2

## Abstract

**Background:**

Growing evidence suggests that Wnt/β-catenin pathway plays an important role in CRC development, progression and metastasis. Aberrant miR-224 expression has been reported in CRC. However, the mechanism of miR-224 promotes both proliferation and metastatic ability largely remains unclear.

**Methods:**

Real-time PCR was used to quantify miR-224 expression. Luciferase reporter assays were conducted to confirm the activity of Wnt/β-catenin pathway and target gene associations, and immunofluorescence staining assay was performed to observe the nuclear translocation of β-catenin. Bioinformatics analysis combined with in vivo and vitro functional assays showed the potential target genes, GSK3β and SFRP2, of miR-224. Specimens from forty patients with CRC were analyzed for the expression of miR-224 and the relationship with GSK3β/SFRP2 by real-time PCR and western blot.

**Results:**

Bioinformatics and cell luciferase function studies verified the direct regulation of miR-224 on the 3’-UTR of the GSK3β and SFRP2 genes, which leads to the activation of Wnt/β-catenin signaling and the nuclear translocation of β-catenin. In addition, knockdown of miR-224 significantly recovered the expression of GSK3β and SFRP2 and attenuated Wnt/β-catenin-mediated cell metastasis and proliferation. The ectopic upregulation of miR-224 dramatically inhibited the expression of GSK3β/SFRP2 and enhanced CRC proliferation and invasion.

**Conclusion:**

Our research showed mechanistic links between miR-224 and Wnt/β-catenin in the pathogenesis of CRC through modulation of GSK3β and SFRP2.

**Electronic supplementary material:**

The online version of this article (doi:10.1186/s13046-016-0287-1) contains supplementary material, which is available to authorized users.

## Background

Human colorectal cancer (CRC) is one of the most common types of malignant tumor worldwide [1]. Although several kinds of treatment modalities have been developed recently for the patients with CRC, the clinical outcome of prognosis continues to be poor in patients with advanced CRC. Metastasis is responsible for the majority of cancer deaths. The aberrant activation of Wnt/β-catenin signaling pathway is considered to be an essential issue in tumorigenesis and progression of CRC [2, 3]. The hallmark of the Wnt/β-catenin pathway is the accumulation and nuclear localization of β-catenin [4, 5]. Cytoplasmic β-catenin is controlled by the degradation complex composed of adenomatosis polyposis coli (APC), Axin, protein phosphatase 2A (PP2A), glycogen synthase kinase 3 beta (GSK3β) and casein kinase 1α (CK1α) [6–9]. When Wnt signaling is activated, β-catenin is discharged from the degradation of complex resulting in the translocation of β-catenin into nucleus, where it associates with the T-cell factor/lymphoid enhancer factor (TCF/LEF) family of transcription factors to activate specific Wnt target genes [10, 11]. It has been well known that inactivating mutation of APC gene has been considered to be an crucial event for constitutive activation of Wnt/β-catenin signaling mutation which leads to carcinogenesis and progression in CRC [12, 13]. However, it has been reported that the mutation of APC cannot fully explain the reason of colorectal tumor carcinogenesis [14, 15]. Thus, alternative mechanisms through which Wnt/β-catenin signaling were activated in CRC might exist.

MicroRNAs (miRNAs) are a class of noncoding small RNAs that play essential roles in the modulation of various biological processes through directly binding to the 3’ untranslated region (3’-UTR) of target genes, which resulting in posttranscriptional inhibition and mRNA cleavage [16, 17]. Accumulating evidence has indicated that dysregulation of miRNAs is closely related to the development and progression of CRC [18, 19]. For example, MicroRNA-30b can function as a tumor suppressor in human colorectal cancer by targeting KRAS, PIK3CD and BCL2 [20]. Also, our previous study has demonstrated that miR-224 can promote cell proliferation by repressing PHLPP1 and PHLPP2 in CRC [21].

In this study, we displayed the critical role of miR-224 in activating Wnt/β-catenin signaling pathway during colorectal progression. We showed that promotion effect of miR-224 on proliferation and invasion of human CRC cells could be manifested partly through the accumulation and nuclear translocation of β-catenin and subsequently up-regulation of its transcriptional targets c-Myc [22, 23] and CyclinD1 [24, 25]. Moreover, we demonstrated that both of Wnt/β-catenin signaling pathway suppressors GSK3β and SFRP2 are bona fide downstream targets of miR-224.

## Methods

### Tissue specimens and cell cultures

The 40 freshly collected CRC specimens and their matched adjacent peri-cancerous normal tissues and distantly metastasis tissues were collected at the operation room, Nanfang Hospital. The fresh specimens were frozen and stored in liquid nitrogen until further use. For the use of clinical materials for research purposes, prior approval was obtained from the Southern Medical University Institutional Board (Guangzhou, China). All samples were collected and analyzed with the prior written, informed consent of the patients.

Two human CRC lines SW480 and HCT116 were purchased from American Type Culture Collection Cell Biology Collection and were maintained in Department of Pathology, Southern Medical University. SW480 and HCT 116 cell lines were cultured in RPMI 1640 (Invitrogen, Carlsbad, CA, USA). All medium was supplemented with 10 % FBS (PAA Laboratories, Pasching, Austria) and 1 % penicillin/streptomycin (Invitrogen) at 37 °C with 5 % CO2.

### RNA isolation, reverse transcription (RT) and real-time PCR

Total RNA from cultured cells and fresh surgical CRC tissues was isolated using the mirVana miRNA Isolation Kit (Ambion) according to the manufacturer’s instruction. The cDNA was then synthesized from total RNA using the Taqman miRNA reverse transcription kit (Applied Biosystems, Foster City, CA, USA). Real-time PCR was performed with the Applied Biosystems 7500 Sequence Detection system, using iQ™ SYBR Green Supermix (BioRad Laboratories, Hercules, CA, USA) containing 5 ng cDNA and 10 pM of each primer. The cycling conditions were set as previously described [20]. The data were normalized to the geometric mean of housekeeping gene GAPDH or U6 small nuclear RNA expression and calculated as 2^−ΔΔCT^ method. Primers for real-time PCR were designed using Primer Premier 5 software. Sequences of the primers are summarized in Additional file 1: Table S1.

### Plasmids and transfection

To generate a miR-224 expression vector, a 281 bp genomic fragment covering the region coding for pri-miR-224 and its upstream and downstream regions was PCR amplified and cloned into the pLvthm vector (Addgene). The fulllength of GSK3β 3’-UTR is 4849 bp long and the SFRP2 3’-UTR is 876 bp long. The miR-224 binding site in the GSK3β 3’-UTR is located at 4755 to 4762 bp, and 316 to 323 bp in the SFRP2 3’-UTR. The region of the human GSK3β 3’-UTR from 4635 to 4825 bp and SFRP2 3’-UTR from 209 to 549 bp were generated by PCR amplification and subcloned into the MluI/NheI sites of the pGL3-basic luciferase reporter plasmid (Promega). The miR-224 mimics, negative control and anti-miR-224 inhibitors were purchased from Genecopoeia (Genecopoeia Co. Ltd.) and transfected into CRC cells using Lipofectamine 2000 reagent (Invitrogen), according to the manufacturer’s instructions. Two concentrations of miR-224 mimics or anti-miR-224 inhibitors (20 and 50 nM) were applied. Also, the CDS of GSK3β was generated by PCR amplification and subcloned into the NheI/EcoRI sites of the pSin-EF2-puro plasmid, and the CDS of SFRP2 was generated by PCR amplification and subcloned into the SpeI/EcoRI sites of the pSin-EF2-puro plasmid. The primers used to generate above constructs are listed in Additional file 2: Table S2. The stable cell lines were established as previously described [26].

### Western blotting

We performed western blot according to the previous study [21]. Protein lysates were prepared, subjected to SDS-PAGE, transferred onto PVDF membranes and blotted according to standard methods, using anti-GSK3β, anti-phospho-β-catenin (Ser9), (Cell Signaling Technology), anti-CyclinD1, anti-c-Myc (Bioworld Technology, St. Louis Park, MN, USA)), anti-Ki-67 (Abcam, Cambridge, MA, USA), anti-MMP7, anti-SFRP2 (BD Biosciences, San Diego, CA, USA), anti-β-catenin (BD Biosciences, San Diego, CA, USA). Anti-α-Tubulin monoclonal antibody (Sigma, St Louis, MO, USA) served as a loading control.

### Luciferase assays

Cells (8 × 10^4^) were seeded in triplicate in 24-well plates and allowed to settle for 24 h. Luciferase reporter plasmids (100 ng) or 100 ng control luciferase plasmid plus 1 ng pRL-TK Renilla plasmid (Promega) were transfected into colorectal cancer cells using Lipofectamine 2000 (Invitrogen). Luciferase and Renilla signals were determined 24 h after transfection using a Dual Luciferase Reporter Assay Kit (Promega) [27, 28].

### Soft-agar colony formation assay, three-dimensional morphogenesis assay, transwell assay and immunohistochemistry

The miR-224 mimics, anti-miR-224 inhibitors, negative control oligos and GSK3β/SFRP2 plasmids were transiently transfected into CRC cells for the soft agar colony-formation assay, three-dimensional morphogenesis assay, transwell assay and immunohistochemistry, as previously described [21, 29].

### Xenograft model in nude mice

For tumourigenesis assays, Xenograft tumors were generated by subcutaneous injection of stable cell lines. Animal experiments were conducted as previously described [20]. Details are as previously Supplementary Materials and Methods described [21]. All mice were obtained from the Animal Center of Southern Medical University, Guangzhou, China, and housed and maintained under specific pathogen-free conditions, and all experiments were approved by the Use Committee for Animal Care and performed in accordance with institutional guidelines. Tumor size was measured using a slide caliper and tumor volume was determined by the formula: 0.44 × A × B^2^, where A represents the diameter of the base of the tumor and B represents the corresponding perpendicular value.

### Statistical analysis

All experiments were performed at least twice. Results are expressed as mean ± S.E.M. where applicable. The two-tailed Student’s *t*-test was used to compare the intergroup. Spearman’s correlation analyses were used to analyze the relationship between miR-224 expression and target genes. Differences between groups were considered statistically significant at *p* < 0.05. Analyses were done with SPSS 19.0 software (IBM, Armonk, NY, USA).

## Results

### MiR-224 activates the Wnt/β-catenin signaling in CRC cells

**Fig. 1:**
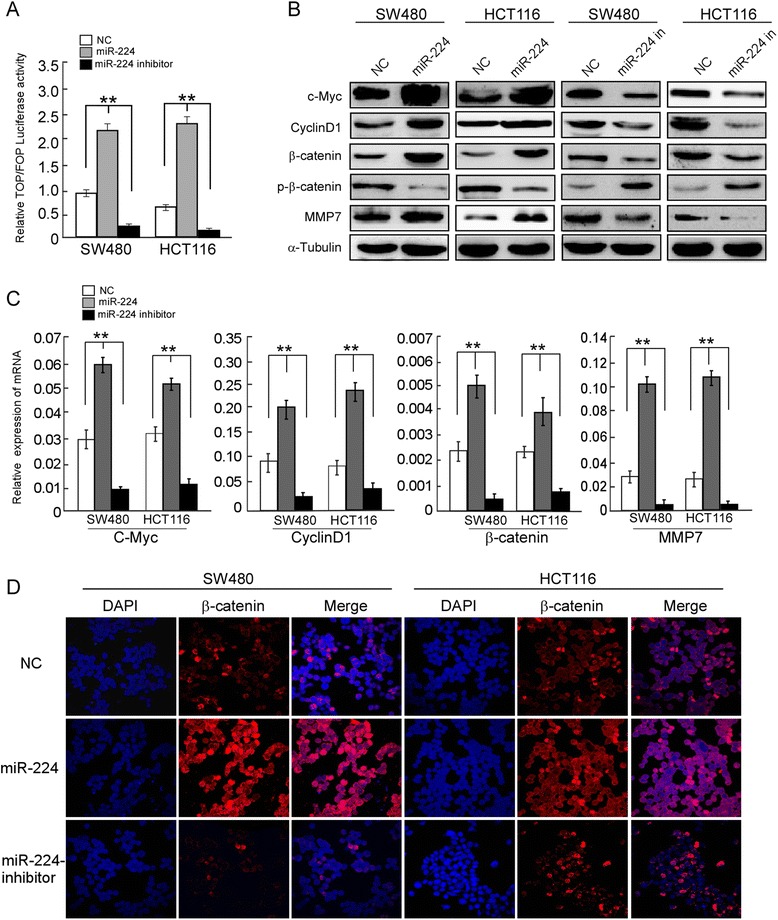
MiR-224 activates the Wnt/β-catenin signaling and results in subsequent β-catenin relocation. **a** TOP/FOP Luciferase assay revealed that the activity of Wnt/β-catenin signaling pathway positively correlated with miR-224 expression. **b** Western blot analysis of crucial downstream molecules and (**c**) real-time PCR analysis of crucial downstream molecules. **d** β-catenin expression was assessed by immunofluorescence staining in both cell lines treated with the negative control miRNA or miR-224 mimics or miR-224 inhibitors for 48 h. β-catenin is shown in red, and the nuclei are counterstained with DAPI that are shown in blue. Scale bars = 10 μm. Error bars represent mean ± SD from 3 independent experiments; * *p* < 0.05, ** *p* < 0.01

Previously, we found that miR-224 could promote cell proliferation by repressing PHLPP1 and PHLPP2 in CRC. Besides the activation of AKT signaling, we also observed that miR-224 can activated Wnt/β-catenin signaling. The activity of Wnt/β-catenin signaling pathway by TOP/FOP Luciferase assay was significantly increased in miR-224-overexpressing CRC cells and reduced in miR-224-inhibited CRC cells (Fig. 1a). Further, we carried out Western blot and real-time PCR to analyze the transcriptional downstream genes of Wnt/β-catenin signaling. Figure 1b and c showed that overexpression of miR-224 significantly upregulated c-Myc, CyclinD1, MMP7 and the activity of β-catenin, while inhibition of miR-224 obviously downregualted c-Myc, CyclinD1, MMP7 and the activity of β-catenin. Moreover, immunofluorescence staining assay displayed that overexpression of miR-224 could evidently promote the nuclear localization of β-catenin (Fig. 1d).

### MiR-224 directly targets GSK3β and SFRP2

**Fig. 2:**
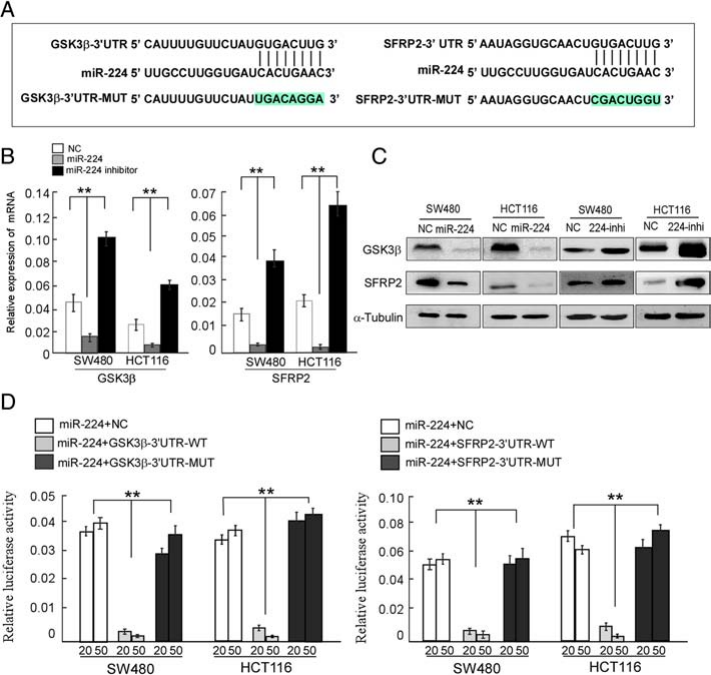
MiR-224 could target GSK3β and SFRP2 in CRC. **a** Predicted miR-224 target sequences in the 3’-UTRs of GSK3β or SFRP2, and their mutants containing altered nucleotides in the 3’-UTRs. **b** Real-time PCR analysis of GSK3β or SFRP2. **c** Western blot analysis of GSK3β or SFRP2. **d** Luciferase assay analyses of the indicated cells transfected with the indicated reporters with increasing amounts of miR-224 (20 and 50 nM). Error bars represent mean ± SD from three independent experiments; * *p* < 0.05, ** *p* < 0.01

In order to predict the putative miR-224 target genes, target prediction program (TargetScan) was applied. The results revealed that GSK3β and SFRP2 are two potential targets of miR-224. The 3’-UTR of GSK3β and SFRP2 mRNA contains a complementary site for the seed region of miR-224 (Fig. 2a). It has been reported that GSK3β and SFRP2 are key regulators of Wnt signaling by controlling the activity of the β-catenin/TCF transcription complex. Real-time PCR (Fig. 2b) and western blot (Fig. 2c) results showed that both the mRNA and protein levels of both GSK3β and SFRP2 were significantly downregulated in miR-224-overexpressing cells, whereas GSK3β and SFRP2 were upregulated after inhibition of miR-224 in CRC cells. Furthermore, we subcloned the wild-type and mutant fragments of GSK3β or SFRP2 3’-UTR fragment separately into the pGL3-basic luciferase reporter vectors. As showed in Fig. 2d, both wild-type GSK3β and SFRP2 reporter gene luciferase activity was reduced upon overexpression of miR-224 in both colorectal cancer cell lines, whereas inhibition of miR-224 increased wild-type GSK3β or SFRP2 luciferase activity.

### MiR-224 promote migration and proliferation of CRC cells partly through inhibition of GSK3β and SFRP2 in vitro

**Fig. 3:**
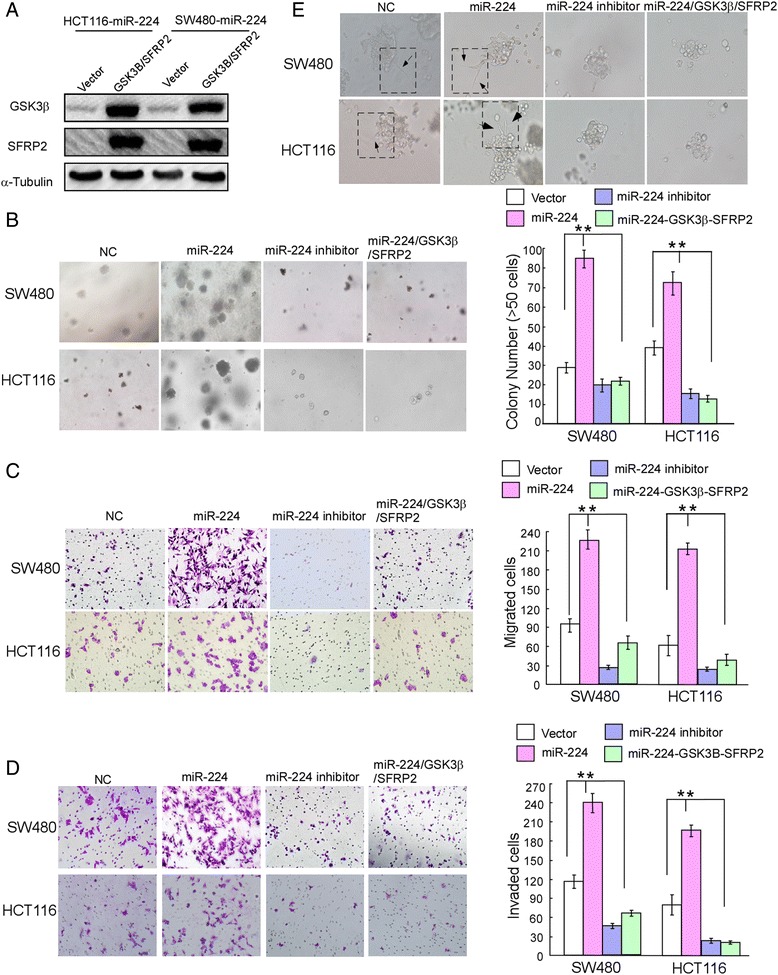
MiR-224 manipulates the colon cancer cells proliferation and migration partly through GSK3β and SFRP2. **a** Western blot analysis of GSK3β or SFRP2. **b** Soft agar assay. Representative micrographs are shown (left) and only cell colonies > 0.1 mm in diameter were counted (right). **c** Representative images (left) and quantification (right) of migrated cells across a Transwell chamber. **d** Representative images (left) and quantification (right) of invaded cells across a Transwell chamber. **e** Representative micrographs of indicated cultured cells at day 7 of culture in three-dimensional morphogenesis assay. Error bars represent mean ± SD from 3 independent experiments; *p < 0.05, ** *p* < 0.01

GSK3β and SFRP2 are important effectors of numerous miRNAs in human cancers, so we need to further explore the biological effects of miR-224 on GSK3β and SFRP2 in a reverse logic. We adopted western blot assay to detected the role of miR-224 in CRC cells under overexpression of GSK3β and SFRP2 (Fig. 3a). Cell function assays displayed that upregulation of miR-224 revealed higher proliferation, migration and invasion capacity, while ectopic up-regulated GSK3β and SFRP2 could partially reverse the influence of miR-224 on CRC cells growth, migration and invasion. Soft agar assays displayed that inhibition of miR-224 evidently reduced the growth rate of both colorectal cancer cell lines while overexpression of miR-224 promoted the growth rate of both colorectal cancer cell lines (Fig. 3b). Impact of miR-224 on cell migration (Fig. 3c) and invasion (Fig. 3d) across a Transwell chamber and three-dimensional morphogenesis assay (Fig. 3e) showed that inhibition of miR-224 supressed migration and invasion capacity of both colorectal cancer cell lines, while overexpression of miR-224 enhanced migration and invasion capacity of both colorectal cancer cell lines. Especially, compared with other cells, overexpression of miR-224 displayed a long spindle-shaped form with thin, long pseudopods, which occasionally resembled finger-like pseudopods that extended from the cell bodies (Fig. 3e). In addition, the results showed ectopic overexprssion of GSK3β and SFRP2 could partially reverse the influence of miR-224 on cell growth migration and invasion.

### GSK3β and SFRP2 are the bona fide effector of miR-224 in vivo

**Fig. 4:**
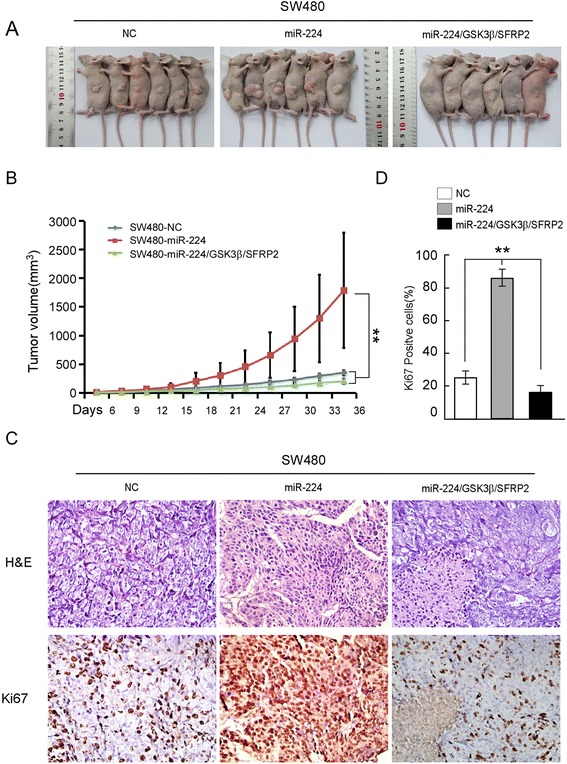
GSK3β and SFRP2 are the bona fide effector of miR-224 in vivo. **a** Tumor xenograft model. Cells were injected into the hindlimbs of nude mice (n = 6). Representative images of the tumors are shown. **b** Tumor volumes were measured on the indicated days. Data points are presented as the mean tumor volume ± SD. **c** Histopathology of xenograft tumors. The tumor sections were under H&E staining and IHC staining using antibody against Ki-67. **d** The percentage of Ki67 positive cells. Error bars represent mean ± SD from 3 independent experiments; * *p* < 0.05, ** *p* < 0.01

To understand whether GSK3β and SFRP2 are involved in miR-224 mediated CRC tumorgenesis in vivo, we engineered SW480 cells to stably overexpress miR-224. The control cells, miR-224-overexpressing cells and the restored GSK3β and SFRP2 in miR-224-overexpressing cells were subcutaneously inoculated into nude mice separately. As shown in Fig. 4a and b, the tumors in the SW480/miR-224 group grew more rapidly than the other group tumors, SW480/NC group and SW480/miR-224-GSK3β -SFRP2 group (*P* < 0.01). IHC staining (Fig. 4c/d) further confirmed that the tumors of the miR-224-overexpressing group displayed much higher Ki-67 indexes than the other two groups.

### Clinical data confirmed GSK3β or SFRP2 are the direct targets of miR-224

**Fig. 5:**
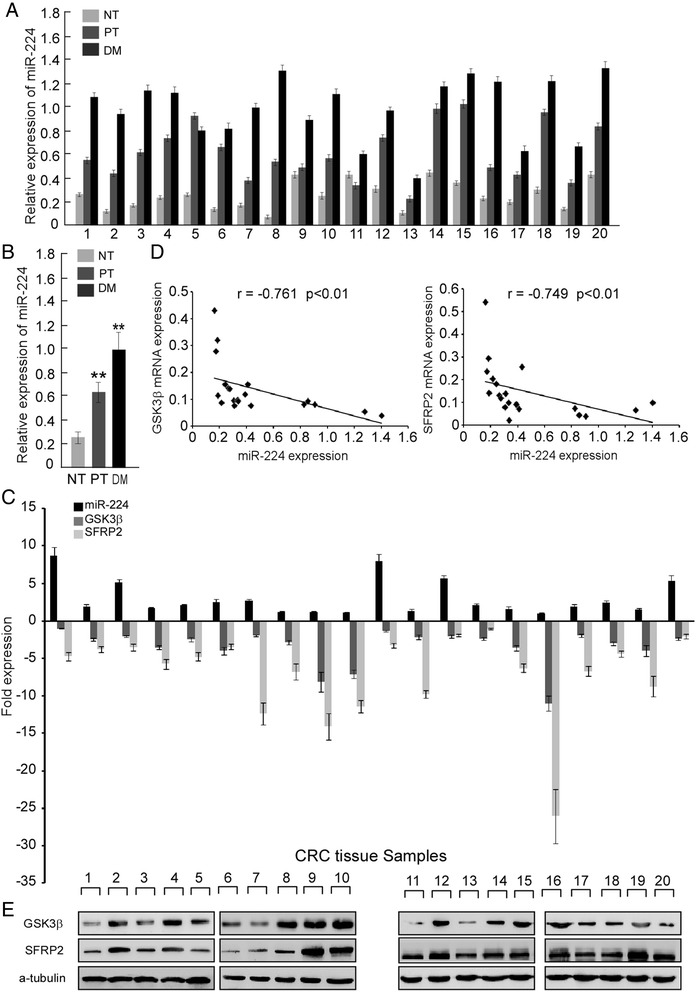
Clinical data confirmed GSK3β or SFRP2 are the direct targets of miR-224. **a** Real-time PCR analysis of miR-224 expression in 20 pairs of peri-cancerous normal tissues (NT), CRC primary tumor tissues (PT), and distantly metastasis tissues (liver, DM); miR-224 expression was normalized to U6. **b** Mean expression of miR-224 in the 20 pairs of peri-cancerous normal tissues (NT), CRC primary tumor (PT), and distantly metastasis (DM). **c** Real-time PCR analysis of miR-224, GSK3β and SFRP2 expression and (**d**) Spearman correlation analyses between relative miR-224 expression and relative mRNA expression levels of GSK3β and SFRP2 in 20 fresh human CRC samples. **e** Western blot analysis of GSK3β and SFRP2 in the 20 fresh human CRC samples; α-Tubulin was used as loading control (lower panel). Error bars represent mean ± SD from 3 independent experiments; * *p* < 0.05, ** *p* < 0.01

To further verify the above observations, miR-224 directly targets GSK3β and SFRP2, could be supported by observations in human CRC. We first tested the expression of miR-224 by real-time PCR analysis in 20 pairs of distant metastasis (liver, DM), CRC primary tumor (PT), and peri-cancerous normal tissues (NT). The results (Fig. 5a and b) revealed that miR-224 was significantly overexpressed in DM and PT tissues than NT tissues (*p* < 0.01). Moreover, the expression levels of miR-224 were higher in DM than in PT tissues (*p* < 0.01). Then we analyzed 20 pairs of freshly CRC tissues and normal tissues to explore the relationship between miR-224 and GSK3β or SFRP2. Figure 5c revealed that miR-224 was up-regulated in CRC tissues while GSK3β or SFRP2 were down-regulated in CRC tissues. The 20 pairs Q-PCR data was attached in Additional file 3: Data S1. Spearman correlation analyses shows that miR-224 expression negatively correlated with the expressions of GSK3β (*r* = –0.761, *p <* 0.01), SFRP2 (*r* = –0.749, *p <* 0.01) (Fig. 5d). Also, western blot analysis confirmed miR-224 expression negatively correlated with the expressions of GSK3β and SFRP2 (Fig. 5e). The above molecular link provides a significant clue for us to figure out the role of miR-224 in the process of CRC Wnt/β-catenin signaling.

## Discussion

MiRNAs are endogenous non-coding RNAs that are related to several key biological processes of tumor, such as tumor initiation, cell proliferation and metastasis which decrease gene expression through binding to the 3’-UTR of target mRNAs [18, 30, 31]. The Wnt/β-catenin signaling pathway has been recognized to play a critical role in regulating oncogenesis and metastasis [17, 32, 33]. Notablely, many evidence approved aberrant activation of Wnt/β-catenin signaling is a hallmark of colorectal tumorigenesis as well as in later stages of invasion and metastasis [2, 18, 34]. Meanwhile, accumulated evidence showed that miRNAs are crucial modulators of Wnt/β-catenin signaling pathway [35–38]. In addition, recent studies demonstrated that miR-224 can promote cell invasion and proliferation in several human cancers [39–41], especially in CRC [21, 42], suggesting its function as an oncogene. However, no further explanations were carried out to illuminate the relationship between miR-224 and Wnt/β-catenin signaling and the mechanism of miR-224-induced CRC invasion. In this study, we demonstrated the role of miR-224 in activating Wnt/β-catenin signaling, and caused the malignant of CRC.

To explore the impact of miR-224 on Wnt/β-catenin signaling pathway, we tested the activity of Wnt/β-catenin signaling pathway by performing TOP/FOP Luciferase assays. The T cell factor (TCF) activity was significantly increased in miR-224 -overexpressing CRC cells, but was decreased in miR-224-inhibited cells. Furthermore, downstream molecules of Wnt signaling and the activity of β-catenin were significantly positive correlated with miR-224. Furthermore, the results demonstrate that miR-224 could promote β-catenin translocate into nucleus.

To further clarify the functional mechanism by which miR-224 activates the Wnt/β-catenin signaling cascade. By bioinformatics prediction (TargetScan) and functional analysis, we identified GSK3β and SFRP2 as direct downstream targets of miR-224. As a crucial member of Wnt/β-catenin signaling modulators, GSK3β plays a central role in the degradation complex and directly depends on the phosphorylation level of β-catenin [43]. GSK3β knockdown promotes several kinds of cancer proliferation and metastasis by promoting β-catenin translocation into nucleus and upregulated oncogene snail expression to promote cancer cells metastasis [44, 45]. SFRP2 is a newly discovered Wnt signaling negative modulators by direct interaction with Wnts [46, 47]. SFRP2 is also downregulated in colorectal cancer and is linked with the EMT as well as proliferation [48, 49]. Collectively, these studies suggest that restoration of the expression or function of the GSK3β and SFRP2 may represent a potential novel therapeutic intervention strategy for CRC. However, precise details of the mechanisms which regulate GSK3β and SFRP2 need further investigation.

Our results confirmed that both GSK3β and SFRP2 were direct targets of miR-224. More specifically, we found that miR-224 significantly downregulated the expression of GSK3β and SFRP2 in CRC cells. Furthermore, we found that GSK3β and SFRP2 restoration could reverse the biological ability of enforced miR-224 expression in vitro and in xenograft models. Interestingly, we found that the miR-224 expression in distantly metastasis tissues was higher than primer tumor tissues and normal tissues by analysis of 20 pairs of clinical tissues. Finally, expression analyses of miR-224, GSK3β and SFRP2 in 20 clinical colorectal cancer tissues revealed significant negative correlations between miR-224 and the expression of GSK3β and SFRP2. All these results supported that miR-224 promoted cell metastasis and proliferation through the Wnt/β-catenin pathway.

## Conclusions

In summary, this study demonstrates meaningful evidence expounding a new functional role of miR-224 in CRC. We showed that miR-224 has the ability to promote CRC cell growth and invasion, which are the basal behavior in CRC metastasis, through directly activating Wnt/β-catenin signaling by targeting GSK3β and SFRP2. Also, restoring the expression of GSK3β and SFRP2 restrained miR-224-induced aggressive phenotype of colorectal cancer cells. Furthermore, miR-224 expression is upregulated in colorectal cancer tumor samples and is inversely correlated with GSK3β and SFRP2. Our results discovered a new role of miR-224, modulating Wnt/β-catenin signaling in CRC tumorgenesis, further indicating that miR-224 may serve as a diagnostic and prognostic biomarker for colorectal cancer.

## Additional files

Additional file 1: Table S1. Primer Sequences Used for Real-time PCR (5' to 3'). (DOC 31 kb) Additional file 2: Table S2. Primer sequences used for amplification and plasmid construction (5′ to 3′). (DOC 30 kb) Additional file 3: Data S1. mRNA expression of miR-224, GSK3βand SFRP2 in 20 CRC tissues. (DOC 15 kb)
