# Correction to: MicroRNA-224 sustains Wnt/β-catenin signaling and promotes aggressive phenotype of colorectal cancer

**DOI:** 10.1186/s13046-021-01945-3

**Published:** 2021-04-27

**Authors:** Tingting Li, Qiuhua Lai, Shuyang Wang, Juanjuan Cai, Zhiyuan Xiao, Danling Deng, Liuqing He, Hongli Jiao, Yaping Ye, Li Liang, Yanqing Ding, Wenting Liao

**Affiliations:** 1 Department of Pathology, Nanfang Hospital, Southern Medical University, Guangzhou, 510515 Guangdong China; 2 Department of Pathology, School of Basic Medical Sciences, Southern Medical University, Guangzhou, Guangdong China; 3 State Key Laboratory of Oncology in Southern China, Department of Experimental, Guangzhou, Guangdong China

**Correction to: J Exp Clin Cancer Res 35, 21 (2016)**

**https://doi.org/10.1186/s13046-016-0287-1**

Following publication of the original article [1], the authors identified minor errors in image-typesetting in Figs. 3 and 4; specifically:

**Fig. 3:**
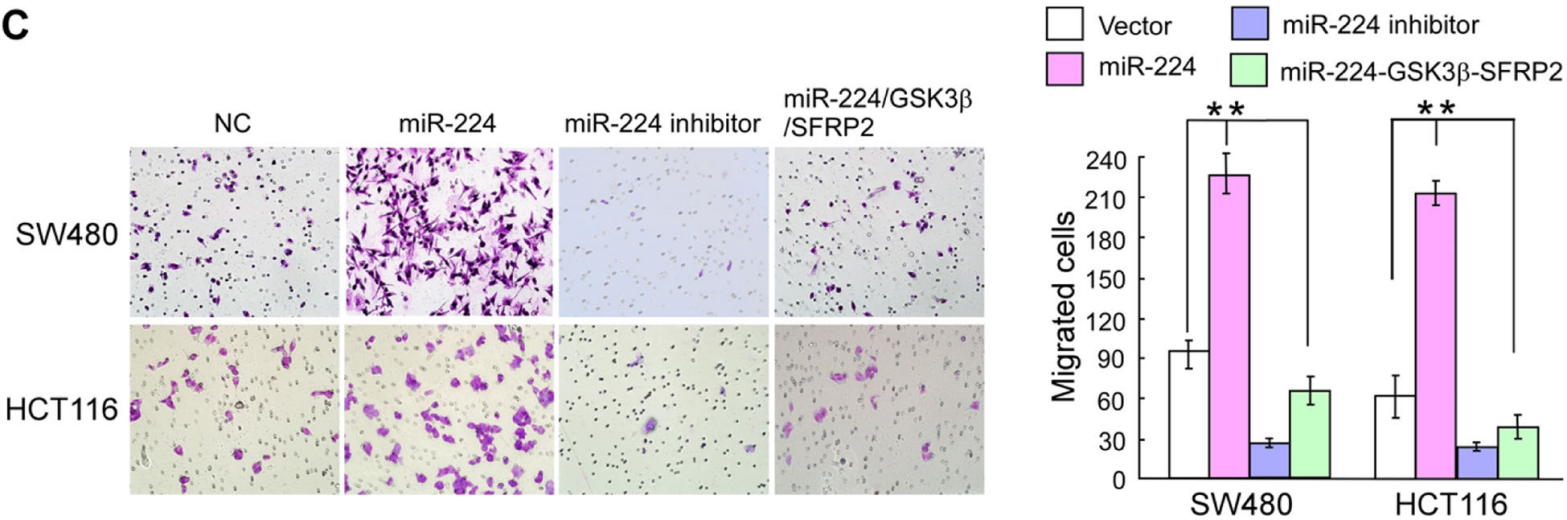
**c** Representative images (left) and quantification (right) of migrated cells across a Transwell chamber

**Fig. 4:**
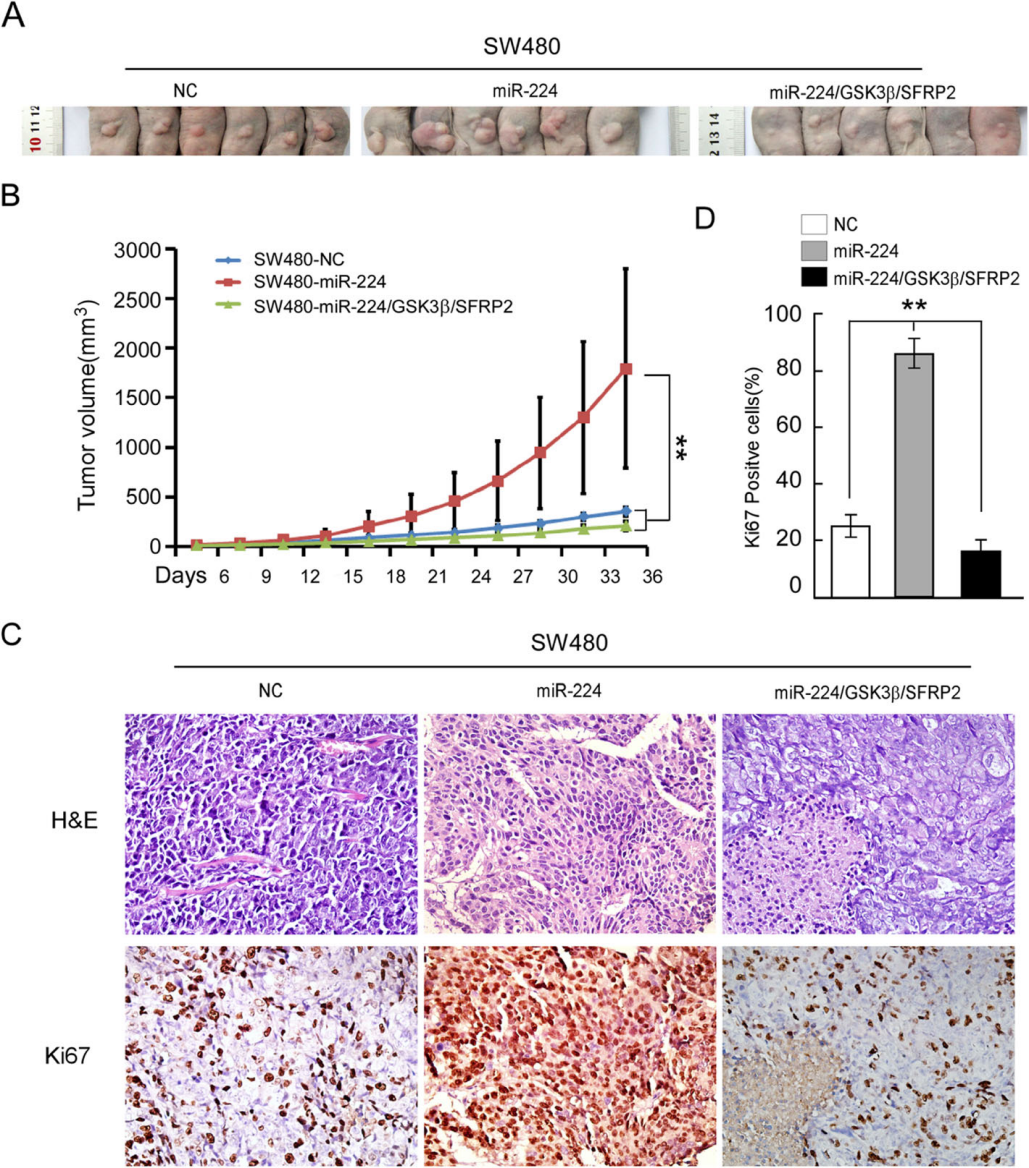
GSK3β and SFRP2 are the bona fide effector of miR-224 in vivo. **a** Tumor xenograft model. Cells were injected into the hindlimbs of nude mice (*n* = 6). Representative images of the tumors are shown. **b** Tumor volumes were measured on the indicated days. Data points are presented as the mean tumor volume ± SD. **c** Histopathology of xenograft tumors. The tumor sections were under H&E staining and IHC staining using antibody against Ki-67. **d** The percentage of Ki67 positive cells. Error bars represent mean ± SD from 3 independent experiments; * *p* < 0.05, ** *p* < 0.01

3C: miR-224/GSK3B/SFRP2 group (top and bottom right panel)

4A: figure adjusted to show only areas of the body containing the tumour

4C: H&E staining in the NC group (top left)

The corrected figures are given below. The corrections do not have any effect on the results or conclusions of the paper. The original article has been corrected.
