# Supplementary material for: MicroRNA-224 sustains Wnt/β-catenin signaling and promotes aggressive phenotype of colorectal cancer
Source: J Exp Clin Cancer Res. 2016 Jan 29;35:21. doi: 10.1186/s13046-016-0287-1 (PMC4731927; doi:10.1186/s13046-016-0287-1)
Supplement: Additional file 1: Table S1. — Primer Sequences Used for Real-time PCR (5' to 3'). (DOC 31 kb) [file 13046_2016_287_MOESM1_ESM.doc]

**Table S1.** Primer Sequences Used for Real-time PCR (5' to 3')

| **Gene** | **Forward primer** | **Reverse primer** |
| --- | --- | --- |
| GAPDH | GACTCATGACCACAGTCCATGC | AGAGGCAGGGATGATGTTCTG |
| CyclinD1 | GCTGCGAAGTGGAAACCATC | CCTCCTTCTGCACACATTTGAA |
| SFRP2 | CATCCAGCCATGCCACTC | GGAAACGGTCGCACTCAA |
| MMP7 | GAGTGAGCTACAGTGGGAACA | CTATGACGCGGGAGTTTAACAT |
| GSK3β | CAACTGCCCGACTAACAC | GAGGAGGAATAAGGATGGTA |
| β-catenin | CATCTACACAGTTTGATGCTGCT | GCAGTTTTGTCAGTTCAGGGA |
| c-Myc | GTCAAGAGGCGAACACACAAC | TTGGACGGACAGGATGTATGC |
