# Supplementary material for: MicroRNA-224 sustains Wnt/β-catenin signaling and promotes aggressive phenotype of colorectal cancer
Source: J Exp Clin Cancer Res. 2016 Jan 29;35:21. doi: 10.1186/s13046-016-0287-1 (PMC4731927; doi:10.1186/s13046-016-0287-1)
Supplement: Additional file 2: Table S2. — Primer sequences used for amplification and plasmid construction (5′ to 3′). (DOC 30 kb) [file 13046_2016_287_MOESM2_ESM.doc]

**Table S2.** Primer sequences used for amplification and plasmid construction (5′ to 3′)

| **Gene** | **Forward primer** | **Reverse primer** |
| --- | --- | --- |
| miR-224 | CACTCCCAAGGAGGTCTGGTGC | CAGCTAACCATGGGCCTGCCT |
| GSK3β-3’  UTR-WT | CGACGCGTCTACTTGAATGCCTCTGTG | GCGCGATCGATTTCCAGGCTAACCTACT |
| SFRP2-3’  UTR-WT | GACGCGTAAAGCCCACCCGAATC | GCGCGATCGGTTCCCATTGAAGGTAT |
| GSK3β-WT | CGCTAGCTATGTCAGGGCGGCCCA | CCGGAATTCAGTCCACCTCAACCTTCGACTA |
| SFRP2-WT | GACTAGTATGCTGCAGGGCCCTGGCT | CCGGAATTCGATCGTGACGTCGAACGC |
