# Supplementary material for: MicroRNA-224 sustains Wnt/β-catenin signaling and promotes aggressive phenotype of colorectal cancer
Source: J Exp Clin Cancer Res. 2016 Jan 29;35:21. doi: 10.1186/s13046-016-0287-1 (PMC4731927; doi:10.1186/s13046-016-0287-1)
Supplement: Additional file 3: Data S1. — mRNA expression of miR-224, GSK3βand SFRP2 in 20 CRC tissues. (DOC 15 kb) [file 13046_2016_287_MOESM3_ESM.doc]

**Data S1.** mRNA expression of miR-224, GSK3βand SFRP2 in 20 CRC tissues

| 0rder | miR-224 | GSK3β | SFRP2 |
| --- | --- | --- | --- |
| 1 | 1.402017±0.164915 | 0.039201±0.003217 | 0.098806±0.010694 |
| 2 | 0.305645±0.04543 | 0.095686±0.00725 | 0.078841±0.007879 |
| 3 | 0.823384±0.062169 | 0.08097±0.004883 | 0.07342±0.008616 |
| 4 | 0.276724±0.015822 | 0.139624±0.009636 | 0.119788±0.014144 |
| 5 | 0.332069±0.026816 | 0.096519±0.011653 | 0.098956±0.011163 |
| 6 | 0.410658±0.050725 | 0.155701±0.019681 | 0.071398±0.007617 |
| 7 | 0.430889±0.042662 | 0.076266±0.004806 | 0.257225±0.031111 |
| 8 | 0.186449±0.015257 | 0.112903±0.011779 | 0.141738±0.021873 |
| 9 | 0.184289±0.016791 | 0.320749±0.05231 | 0.293149±0.0367 |
| 10 | 0.172356±0.017072 | 0.278386±0.022901 | 0.23742±0.016108 |
| 11 | 1.274561±0.147637 | 0.053201±0.003998 | 0.067638±0.006088 |
| 12 | 0.214053±0.037544 | 0.085863±0.011802 | 0.20395±0.010877 |
| 13 | 0.905722±0.071523 | 0.081087±0.008325 | 0.039642±0.002966 |
| 14 | 0.33621±0.030432 | 0.093207±0.008748 | 0.020833±0.00223 |
| 15 | 0.263072±0.040444 | 0.141476±0.014919 | 0.131427±0.011643 |
| 16 | 0.160251±0.020229 | 0.430908±0.041664 | 0.543069±0.074714 |
| 17 | 0.310425±0.041772 | 0.077319±0.006877 | 0.139428±0.014328 |
| 18 | 0.391542±0.043549 | 0.117219±0.0106 | 0.091872±0.007964 |
| 19 | 0.241564±0.03191 | 0.156185±0.026622 | 0.181511±0.028467 |
| 20 | 0.856211±0.12107 | 0.093747±0.007396 | 0.042883±0.006083 |
